# Circulating antibodies against *Plasmodium falciparum* histidine-rich proteins 2 interfere with antigen detection by rapid diagnostic tests

**DOI:** 10.1186/1475-2875-13-480

**Published:** 2014-12-06

**Authors:** Mei-Fong Ho, Joanne Baker, Nelson Lee, Jennifer Luchavez, Frédéric Ariey, Sina Nhem, Wellington Oyibo, David Bell, Iveth González, Peter Chiodini, Michelle L Gatton, Qin Cheng, James S McCarthy

**Affiliations:** QIMR Berghofer Medical Research Institute, Brisbane, Australia; Department of Drug Resistance and Diagnostics, Australian Army Malaria Institute, Brisbane, Australia; School of Population Health, University of Queensland, Brisbane, Australia; Research Institute for Tropical Medicine, Alabang, Metro Manila, The Philippines; Pasteur Institute of Cambodia, Phnom Penh, Cambodia; College of Medicine, University of Lagos, Odoaraba, Lagos, Nigeria; Foundation for Innovative New Diagnostics (FIND), Geneva, Switzerland; Hospital for Tropical Diseases, London, UK; 6GGIV Unit, Parasitology and Mycology Department, Institute Pasteur, Paris, France; Global Good Fund/Intellectual Ventures Laboratory, Bellevue, WA USA; QUT University, Kelvin Grove, Brisbane, Australia

## Abstract

**Background:**

Rapid diagnostic tests (RDTs) for detection of *Plasmodium falciparum* infection that target *P. falciparum* histidine-rich protein 2 (PfHRP2), a protein that circulates in the blood of patients infected with this species of malaria, are widely used to guide case management. Understanding determinants of PfHRP2 availability in circulation is therefore essential to understanding the performance of PfHRP2-detecting RDTs.

**Methods:**

The possibility that pre-formed host anti-PfHRP2 antibodies may block target antigen detection, thereby causing false negative test results was investigated in this study.

**Results:**

Anti-PfHRP2 antibodies were detected in 19/75 (25%) of plasma samples collected from patients with acute malaria from Cambodia, Nigeria and the Philippines, as well as in 3/28 (10.7%) asymptomatic Solomon Islands residents. Pre-incubation of plasma samples from subjects with high-titre anti-PfHRP2 antibodies with soluble PfHRP2 blocked the detection of the target antigen on two of the three brands of RDTs tested, leading to false negative results. Pre-incubation of the plasma with intact parasitized erythrocytes resulted in a reduction of band intensity at the highest parasite density, and a reduction of lower detection threshold by ten-fold on all three brands of RDTs tested.

**Conclusions:**

These observations indicate possible reduced sensitivity for diagnosis of *P. falciparum* malaria using PfHRP2-detecting RDTs among people with high levels of specific antibodies and low density infection, as well as possible interference with tests configured to detect soluble PfHRP2 in saliva or urine samples. Further investigations are required to assess the impact of pre-formed anti-PfHRP2 antibodies on RDT performance in different transmission settings.

**Electronic supplementary material:**

The online version of this article (doi:10.1186/1475-2875-13-480) contains supplementary material, which is available to authorized users.

## Background

Malaria rapid diagnostic tests (RDTs) are lateral-flow devices that use antibodies to capture and detect parasite proteins by immunochromatography. They have similar sensitivity to light microscopy, are easy to use, do not require sophisticated equipment or electricity, and usually produce results within 20 minutes. They are recommended by the World Health Organization (WHO) as point-of-care diagnostic tools [1] as they provide a parasite-based diagnostic alternative to conventional light microscopy. RDTs are playing an increasingly important role in malaria case management, particularly in areas where good-quality microscopy is not available, with approximately 205 million used globally in 2012 [2]. Indeed, the advent of RDTs has made possible the recent update of WHO guidelines for management of malaria requiring a parasitological diagnosis in all cases [1].

Currently, over 150 malaria RDT brands are commercially available. All utilize antibodies to detect one or more of three parasite proteins: *Plasmodium falciparum* histidine-rich protein 2 (PfHRP2) unique to *P. falciparum,* plasmodium lactate dehydrogenase (pLDH) and aldolase, the latter two being targets for infection with both *P. falciparum* and non-*P. falciparum* species. While malaria RDTs have been reported to have detection sensitivity comparable to that of thick film microscopy, their performance can vary. Although most reports of imperfect sensitivity are at relatively low parasite densities [3–7], false negative results at relatively high parasite densities have also been reported [8, 9]. Possible explanations for imperfect sensitivity at high parasite density include deletion of the *pfhrp2* gene [10], varying quantity of proteins produced by different parasites [11], the prozone effect [12, 13], the performance characteristics of the capture and detection antibodies in the kit, including their thermal stability [14, 15], as well as manufacture quality.

With respect to quality of manufacture, product testing and lot testing carried out by WHO and Foundation for Innovative New Diagnostics (FIND) have demonstrated significant variation in performance between different products in detecting diluted field parasites [16]. These test results provide explanation for poor performance of some RDTs in the field, particularly in detecting moderate and low parasite densities.

A factor that has not been systematically investigated is the effect of antibodies specific for the parasite target antigens that have been generated against these antigens by previous and/or current malaria infections. Such antibodies could bind these circulating antigens and form immune complexes whilst in circulation or when a blood sample is lysed on an RDT, thereby interfering with the binding of antigen to antibodies on the RDT test lines. It is well recognized that many proteins released by the malaria parasite during blood stage infection, including PfHRP2 are immunogenic and generate an antibody response. PfHRP2 accumulates in the parasite cytosol, and within the cytosol of infected red cells [17]. It has been reported to be both released by infected red cells into the blood, as well as following red cell rupture at schizogony [18]. A factor that may favour development of anti-PfHRP2 antibodies is the relatively long half-life of PfHRP2, compared to other parasite proteins such as LDH, with reports that PfHRP2 can circulate for two to four weeks after cure of infection [19–21].

Biswas and colleagues reported a longitudinal follow-up of PfHRP2 antigenaemia and antibody responses in a group of 45 blood smear-positive malaria subjects with *P. falciparum* [22]. They observed that the titre of anti-PfHRP2 antibody gradually rose over the 28 days of follow-up. Three of the 45 samples were RDT test negative at baseline. All three had significantly lower PfHRP2 antigenaemia and higher titres of anti-HRP2 antibodies compared to the 42 RDT-positive samples. This work supports the hypothesis that some malaria-infected individuals may test PfHRP2 negative because all available PfHRP2 is complexed with pre-formed host antibody, and therefore is unavailable for binding with signal antibody on an RDT.

In this study the sero-epidemiology of anti-PfHRP2 antibodies in malaria-endemic populations was investigated and the effect of pre-formed antibodies on RDT performance was explored. The prevalence and titre of anti-PfHRP2 antibodies in a high transmission area of Solomon Islands was investigated, as well as in patients with acute *P. falciparum* malaria in Cambodia, Nigeria and the Philippines. The potential for these antibodies to interfere with the performance of RDTs using both recombinant PfHRP2 and cultured parasites was also investigated.

## Methods

### Cloning, expression and purification of recombinant PfHRP2 (rPfHRP2)

*Pfhrp2* genes originating from *P. falciparum* isolate FCQ79 was cloned into the pET8c expression vector (Novagen), expressed in *Escherichia coli* BL21(DE3) LysS cells and purified using His-trap Ni-IDA column (GE Healthcare) followed by Superdex 75 10/300 column (GE Healthcare) (Lee *et al.,* unpublished).

### Determination of rPfHRP2 concentration

The concentrations of the purified rPfHRP2 were determined using the Biruet assay [23]. Briefly, a working reagent was prepared by mixing an equal volume of stock reagent (5% sodium citrate, 3% sodium carbonate and 0.5% copper sulphate) with 150 mM NaOH. In each well, 40 μl of rPfHRP2 sample was mixed with 160 μl of working reagent in triplicate and incubated at room temperature for 25 min. The absorbance of each well was read at 540 nm and sample concentrations were determined from the standard curve of bovine serum albumin (BSA).

### Collection of human plasma

A total of 121 plasma samples were collected from malaria-endemic regions of four countries at different times. Some were collected as part of the parasite collection for the Specimen Bank used in the WHO Product Testing for malaria RDTs [24, 25]. Samples from acute malaria patients (symptomatic and microscopy positive) were collected at time of presentation from Cambodia (KH, n = 15), Nigeria (NG, n = 23) and the Philippines (PH, n = 37) by Pasteur Institute of Cambodia, University of Lagos and Research Institute for Tropical Medicine, the Philippines [24, 25]. An additional ten and eight samples were collected from microscopy-negative subjects in Nigeria and the Philippines (uninfected endemic controls), respectively. The collection and use of the samples was approved by the ethics committees of the collection institutions. Methods used for blood collection, parasite speciation and quantitation were described in detail in a WHO Methods Manual [26]. Plasma was separated from each patient blood and stored at −20°C. A total of 28 plasma samples were collected in 1987 [27] in Solomon Islands (SB) with informed consent from asymptomatic villagers (13–66 years old) living in Guadalcanal, Solomon Islands [27]. Eleven of these villagers were microscopy positive for *P. falciparum* with low densities while the remaining 17 villagers were microscopy negative at the time of collection. All plasma samples were stored at −80°C. Plasma samples were also collected from six Brisbane residents who had no exposure to *Plasmodium* infection for use as non-exposed negative controls.

Patient blood sample collection was coordinated by WHO, TDR and FIND and conducted by investigators and institutions within the WHO-FIND Malaria RDT Quality Assurance Programme.

### ELISA determination of the level of anti-PfHRP2 antibodies

Purified rPfHRP2 (50 ng/well) was coated on flat-bottomed, 96-well microtitre plates (Nunc, Denmark) at 4°C overnight or 37°C for two hours. Plates were blocked with 1% skim milk at 37°C for one hour. Plasma samples were diluted to 1:200, added in duplicate at 50 μL/well and incubated at 37°C for one hour. Plates were washed five times with wash buffer and air-dried. Anti-human IgG conjugated with horseradish peroxidase (Sigma) was diluted 1:5,000 and added into the wells (50 μL/well). Plates were incubated for one hour at 37°C and then washed five times. OPD substrate buffer (Sigma Aldrich) was added to each well (100 μL/well), and incubated in the dark for 20 minutes at room temperature. Absorbance was read at 450 nm using a microplate reader (VeraMax, Molecular Devices, USA).

To establish relative antibody concentrations against rPfHRP2, five human plasma that contained high anti-PfHRP2 antibody levels were identified and pooled for use as a reference standard. This standard plasma pool was two-fold diluted in PBS from 1:100 to 1:1,600, and applied, in duplicate, to each assay plate. ELISA units were arbitrarily assigned to each dilution. Two wells that contained PBS were used as a negative control and were also used to blank all samples and standard plasma.

The OD_450_ readings of the serial dilutions of the standard plasma pool were plotted on a four-parameter hyperbolic curve (SOFTmax Pro ver 3.1; Molecular Devices). If R^2^ was less than 0.9, the assay was deemed invalid and was repeated. ELISA units in test plasma were interpolated from their mean OD_450_ values from the standard curve. The positive threshold (13.1) was defined as two standard deviations above the mean ELISA units (8.4) of a pool of six human plasma samples from individuals never exposed to malaria.

### Determining the quantity of circulating PfHRP2 antigen in plasma using ELISA

A commercially available SD Malaria Antigen *Pf* ELISA kit (Standard Diagnostics Inc, Korea) was used to detect PfHRP2 antigen in human plasma. The assay was performed following manufacturer’s instructions, with the exception of the initial lysis and conjugation step. Briefly, 50 μL of each sample were incubated with 75 μL of lysis buffer, containing horseradish peroxidase enzyme conjugate, at 37°C for 15–30 minutes. A 100-μL volume of the mixture was then transferred to the supplier’s PfHRP2-coated microtitre plate, and incubated at 37°C for 90 minutes. Plates were washed six times with wash buffer before substrate was added. The reaction was stopped with stopping solution after incubation at room temperature for 30 minutes, and plates were immediately read on a microplate reader (VeraMax, Molecular Devices) at 450 nm with a reference of 620 nm.

To quantify the plasma PfHRP2 level, a PfHRP2 antigen standard concentration curve was established using a set of doubling dilutions of a *P. falciparum* 3D7 culture supernatant. The concentration of PfHRP2 in the culture supernatant (ng/mL) was determined by comparing its OD_450_ value to a known protein concentration standard curve that was prepared by serial dilutions of rPfHRP2 (FCQ79). These standard dilutions were used as a reference standard for each assay plate. The PfHRP2 concentration (ng/mL) in each plasma sample was determined by reference its OD_450_ value to the standard curve assayed on the same plate. The limit of detection of this ELISA was determined to be 0.5 ng/mL.

### Measuring the blocking effects of plasma on the detection of soluble PfHRP2 using ELISAs and RDTs

Two high-titre plasma samples from Solomon Islands, S14 and S53, were identified and used as sources of anti-PfHRP2 antibody; culture supernatant from an *in vitro* culture of the *P. falciparum* strain 3D7 was used as source of parasite PfHRP2 antigen. The culture supernatant was subject to two-fold doubling dilutions with RPMI media from 1:2 to 1:126; 50 μL of each dilution was mixed with 50 μL of plasma, undiluted or diluted 1:5. Culture supernatant was used as the ‘no plasma’ control; pooled plasma from non-exposed negative controls was used as the negative plasma control. Antibody-antigen mixtures were incubated at 37°C for one hour, transferred to the SD Malaria Antigen *Pf* ELISA kit, and processed as described above. The OD_450_ in mixtures with and without human plasma was compared. Concurrently, 5 μl of each mixture was transferred onto three different malaria RDTs: ICT Malaria Combo Cassette Test (ML02, ICT Diagnostics, South Africa), First Response Malaria Antigen pLDH/HRP2 Combo (116FRC30, Premier Medical Corp, India) and SD Malaria Antigen *Pf* (05FK50, Standard Diagnostics, Korea). Results were read by two independent readers and recorded as levels 0–4 based on the test band intensity following the WHO colour chart [24].

### Investigating the blocking effects on ELISA and RDT detection of *Plasmodium falciparum*in tissue culture using plasma containing anti-PfHRP2 antibodies

Three culture-adapted *P. falciparum* lines originating from Solomon Islands (S55, SJ44 and SJ15) were cultured *in vitro* using standard culture conditions [28]. Parasite cultures were repeatedly synchronized using 5% sorbitol [29], then maintained and harvested when parasitaemia reached 2% with majority at ring stage. An aliquot of the harvested parasites was concentrated to 50% haematocrit by centrifuging and discarding excess supernatant. A second aliquot of the culture was pelleted, washed and resuspended in fresh media to a 50% haematocrit. Both aliquots were diluted using normal human blood maintaining a 50% haematocrit to parasite densities of 100,000, 30,000, 10,000, 3,000, 1,000, 300, 100, and 30 parasites/μL. An aliquot of each dilution was incubated with the immune human plasma (S14 or S53) or negative control plasma for one hour at room temperature. These samples were then tested on a RDT as described above.

### Statistical analysis

The ages, parasite densities and PfHPR2 antigen levels in the patient samples were compared between countries of origin using the Kruskal Wallis test. Proportions were compared between countries using either Fisher’s exact test (for comparison of two categories) or Chi-square Test (>two categories). Correlation between parasitaemia and antibody or antigen level was assessed using Spearman’s Rank Correlation.

## Results

### Demographic and parasitologic characteristics of the subjects from whom the plasma samples were collected

Blood samples were collected from patients with acute malaria in Cambodia (n = 15), Nigeria (n = 23) and the Philippines (n = 37). The age of these individuals and levels of parasitaemia are presented in Table 1 and Additional file 1. There were no significant differences in the ages of the patients recruited at each site (P > 0.50). The geometric mean parasite density of samples differed by country of origin (P < 0.001); densities from Nigeria (18,528/μL, 95% CI: 12,686-27,059/μL) and Cambodia (20,940/μL, 95% CI: 9,766-44,897/μL) were not significantly different (P > 0.05), but were significantly higher than in samples from the Philippines (1,767/μL, 95% CI: 811–3,850/μL; P < 0.01). The Solomon Islands samples were collected in 1987 from a high transmission area. All subjects were asymptomatic at the time of collection. Eleven of the 28 individuals were microscopy-positive for *P. falciparum,* with low-density infection (exact parasite density was not available). The remaining 17 individuals, nine of whom reported a history of malaria were blood smear negative at the time of collection.

**Table 1:** **Demographics and parasitaemia of the plasma samples**

Sample type	Country	n	Age		Parasitemia	
			Median	(Range)	Geometric mean	(Range)
Acute patients (symptomatic & microscopy + ve)	Cambodia	15	21	(8–51)	20,940	(2,005-195,665)
	Nigeria	23	23	(9–37)	18,528	(5,118-142,696)
	Philippines	37	19	(4–71)	1,767	(30–79,821)
Asymptomatic (microscopy + ve or –ve)	Solomon Islands	28	*29	(13–66)	11 were microscopy positive for *P. falciparum* with low densities	
Uninfected endemic controls	Nigeria	10	Unknown		N/A	
	Philippines	8	28	(7–76)	N/A	
Non-exposed control	Brisbane	6	Unknown		N/A	

*Calculated based on information available from 17 subjects. An additional five subjects were adults, and six subjects had unknown age.

### Anti-PfHRP2 antibody levels in plasma samples

Plasma samples were analysed for anti-rPfHRP2 antibody levels using ELISA. The mean ELISA unit among the non-exposed negative controls was 8.4 ± 2.3, while the corresponding level for the ten parasite-negative Nigerian samples was 2.7 ± 1.1. Nineteen of the 75 patients with acute malaria (25.3%) had anti-PfHRP2 antibody levels above the cut-off value (13.0 units), with the prevalence of antibodies in samples from Cambodia, Nigeria and the Philippines not being significantly different (20.0, 34.7 and 21.6%, respectively; P > 0.40). The prevalence of anti-PfHRP2 antibodies among the asymptomatic Solomon Islanders was lower at 10.7%, although not significantly different from prevalence in patients with acute malaria (P > 0.15).

**Figure 1:**
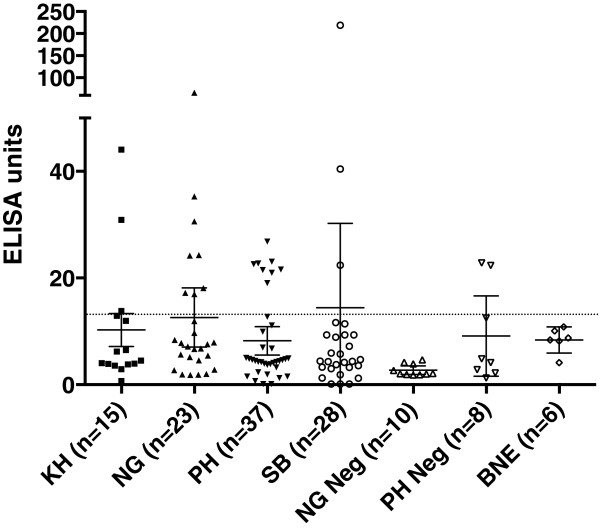
**Anti-PfHRP2 antibody levels in plasma samples.** Levels of PfHRP2-specific antibodies in the plasma of subjects were measured by ELISA. Cut-off is shown by the dotted line, and Error Bars represent 95% Confidence Intervals. Country of origin is represented using ISO 3166 country code: Cambodia (KH), Nigeria (NG), the Philippines (PH), and Solomon Islands (SB). BNE represents Brisbane. Samples from subjects who were microscopy negative for *P. falciparum* are shown as Neg.

Levels of anti-PfHRP2 antibodies are shown in Figure 1 (with raw Optical Density results shown in Additional file 2). The majority of the antibody-positive samples had antibody levels between one to three-fold higher than the cut-off value, while two plasma samples had antibody levels that were five-fold (NG02F22) and 16.7-fold (SB014) higher than the cut-off value. Overall there was a positive correlation between antibody level and age of infected subjects. However, this was not statistically significant for the combined set of parasite positive samples (r = 0.20, P = 0.08, n = 79), nor within individual countries (KH: r = 0.23, P = 0.39; PH: r = 0.05, P = 0.75; NG: r = 0.16, P = 0.47, SB (Pf positive): r = −0.32, P = 0.50). There was also no significant correlation between parasitaemia and antibody level in the symptomatic patient samples (r = −0.06, P = 0.62.)

### PfHRP2 antigen levels in plasma samples

PfHRP2 antigen was detected by ELISA in 100, 91.3 and 62.2% plasma of patients with symptomatic malaria in Cambodia, Nigeria and the Philippines, respectively, while only 6/28 (21.43%) of plasma from asymptomatic subjects from the Solomon Islands had detectable PfHRP2 antigen (Table 2). Five of these six subjects were positive for *P. falciparum* by microscopy. None of the parasite-negative subjects from Nigeria (n = 10), the Philippines (n = 8) or Brisbane (n = 6) had detectable PfHRP2 in their plasma samples (Table 2).

**Figure 2:**
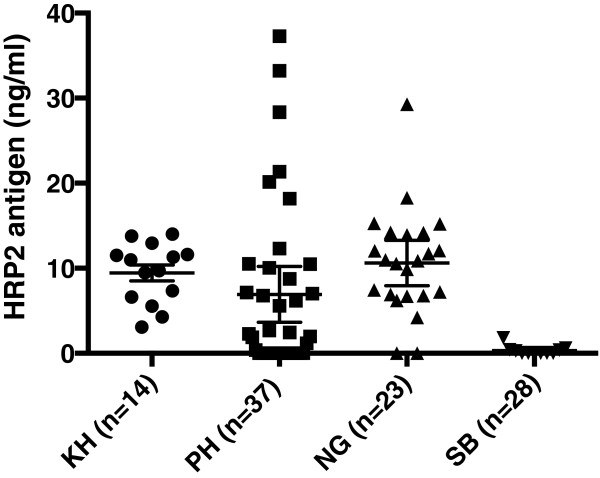
**PfHRP2 antigen levels in plasma samples.** PfHRP2 antigen levels were measured in a commercial ELISA test (Standard Diagnostics, Korea). Country of origin is represented using ISO 3166 country code: Cambodia (KH), Nigeria (NG), the Philippines (PH), and Solomon Islands (SB). Shown are the results from subjects who tested positive. Error Bars represent SEM.

**Figure 3:**
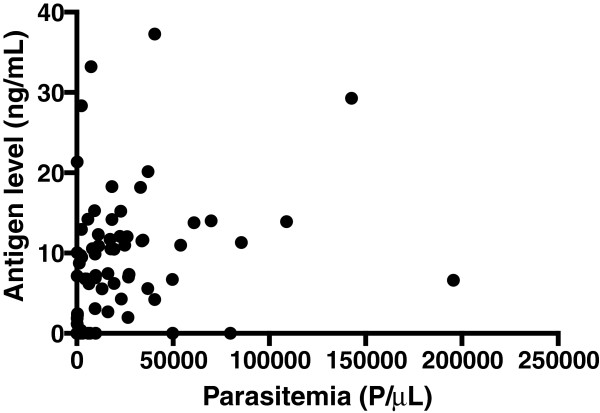
**Relationship between parasitaemia and plasma PfHRP2 level in samples from symptomatic patients.**

The level of PfHRP2 antigen present in individual plasma samples is shown in Figure 2. For samples from symptomatic patients with detectable antigen, there was no significant difference in the distribution of antigen level according to country of origin (P = 0.27). The mean antigen levels were 9.45 ng/mL (95% CI: 7.42-11.49 ng/mL), 11.62 ng/mL (95% CI: 9.14-14.11 ng/mL) and 11.14 ng/mL (95% CI: 6.60-15.69 ng/mL), for Cambodia, Nigeria and the Philippines, respectively. The antigen levels among the Solomon Islands asymptomatic subjects (for which antigen was detected) were significantly lower (mean = 0.70 ng/mL, 95% CI: −0.09-1.48 ng/mL) than for the symptomatic patients in Cambodia, Nigeria and the Philippines (P < 0.01). There was a significant positive correlation between parasite density as determined by blood smear and plasma PfHRP2 level for the symptomatic patient samples (r = 0.45, P < 0.0001, n = 74, Figure 3).

**Table 2:** **Number and proportion of samples positive for PfHRP2 antigen and anti-PfHRP2 antibody by ELISA**

Sample type	Country	n	Antigen	Antibody		
			No	(%)	No	(%)
Acute patients (symptomatic & microscopy + ve)	Cambodia	15	14*	(100)*	3	(20.0)
	Nigeria	23	21	(91.3)	8	(34.7)
	Philippines	37	23	(62.2)	8	(21.6)
	**Sub-total**	**75**	**57**	**(76.0)**	**19**	**(25.3)**
Asymptomatic (microscopy + ve or –ve)	Solomon Islands	28	6	(21.4)	3	(10.7)
Uninfected endemic controls	Nigeria	10	0	0	0	0
	Philippines	8	0	0	2	(25)
Non-exposed controls	Brisbane	6	0	0	0	0

*Antigen measured for 14 samples.

### Correlation between anti-PfHRP2 antibody levels and levels of PfHRP2 antigen

**Figure 4:**
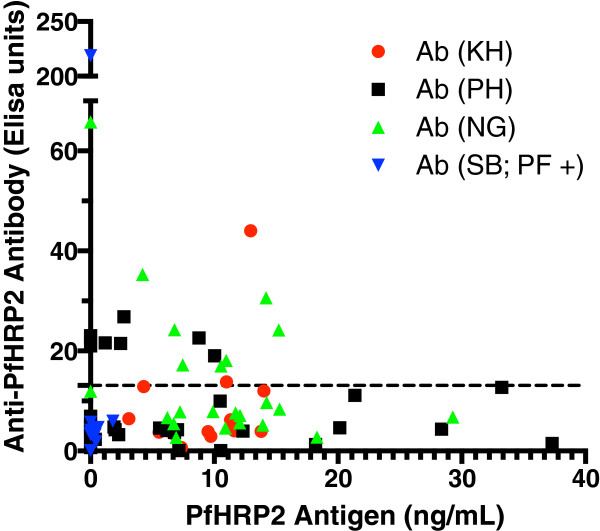
**Relationship between plasma anti-PfHRP2 antibody and plasma PfHRP2 antigen level.** Country of origin is represented using ISO 3166 country code: Cambodia (KH), Nigeria (NG), the Philippines (PH), and Solomon Islands (SB). Ab represents antibody. PF + and PF – represents microscopy positive and negative, respectively, for *P. falciparum*.

Data on anti-PfHRP2 antibody level and PfHRP2 antigen level were available for 74 samples. Of the 18 samples from patients with acute malaria who had anti-PfHRP2 antibodies above the cut-off, 14 (77%) were PfFHRP2 antigenaemic. There was no significant association between antibody presence and presence of antigen (P > 0.90). Logistic regression indicated that antigen level was not a significant factor for predicting the presence of anti-PfHRP2 antibody (P = 0.44). Although samples with high antigen levels tended to have lower antibody levels, and samples with the highest antibody level tended to have low antigen levels (Figure 4), this relationship was not statistically significant.

### The blocking effect of plasma-containing anti-PfHRP2 antibodies on the detection of soluble PfHRP2 and parasitized erythrocytes by RDTs

**Figure 5:**
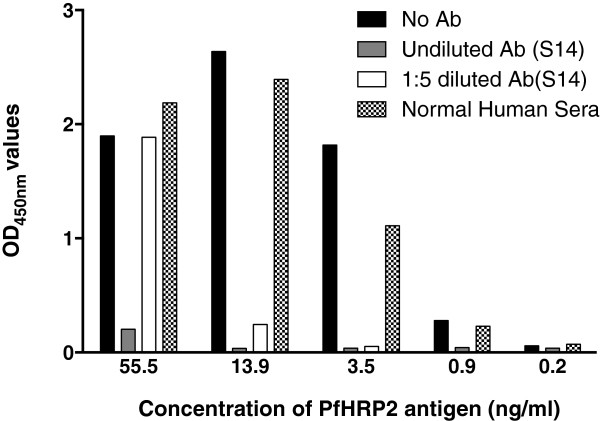
**Anti-PfHRP2 antibody inhibits ELISA detection of soluble PfHRP2 antigen.** Culture supernatant from an *in vitro* culture of *P. falciparum* was pre–incubated in serial dilution with a plasma sample with high-titre anti-PfHRP2 antibody and tested in a commercial HRP2 antigen-capture ELISA. Four-fold dilutions are shown.

When two plasma samples with high-titre anti-PfHRP2 antibody were pre-incubated with serial dilutions of culture supernatant from an *in vitro* culture of the *P. falciparum*, a significant reduction in OD values in the HRP2 antigen-capture ELISA was observed at all antigen dilutions tested. Figure 5 demonstrates the effect of a serum from one of these two subjects (S14). The same effect was also observed when the plasma was diluted five-fold, except at the highest antigen concentration tested (55.5 ng/mL), or when undiluted plasma from another subject with high-titre antibody (S53) was used. The plasma pool from Brisbane subjects had no or minimal effect on the detection of antigens by ELISA.

When the pre-mixed plasma-antigen mixtures were tested on three commercially available malaria RDTs, complete blocking of the detection was observed with two brands of RDT (First Response and SD) resulting in negative RDT results (Table 3). The blocking effect diminished when the plasma was diluted five-fold. Interestingly, the performance of the third brand of RDT (ICT combo) was not as markedly affected by pre-incubation with plasma with high-titre, anti-PfHRP2 antibody, and tested positive in all situations (Table 3).

**Table 3:** **The effect of anti-PfHRP2 antibodies on the detection of soluble PfHRP2 by RDTs**

Culture supernatant	First response				SD				ICT			
	No Ab	Normal plasma	S14	S14 1:5	No Ab	Normal plasma	S14	S14 1:5	No Ab	Normal plasma	S14	S14 1:5
Undiluted	4	4	0	1	4	4	0	2	4	4	4	4
1:2	4	4	0	0	4	4	0	0	4	3	3	2
1:4	4	2	0	0	3	3	0	0	3	2	1	1
1:8	1	1	0	0	1	2	0	0	2	1	1	1
1:16	1	1	0	0	1	1	0	0	1	1	1	1
1:32	0	0	0	0	0	0	0	0	1	0	0	0
1:64	0	0	0	0	0	0	0	0	1	0	0	0
1:128	0	0	0	0	0	0	0	0	1	0	0	0

**Figure 6:**
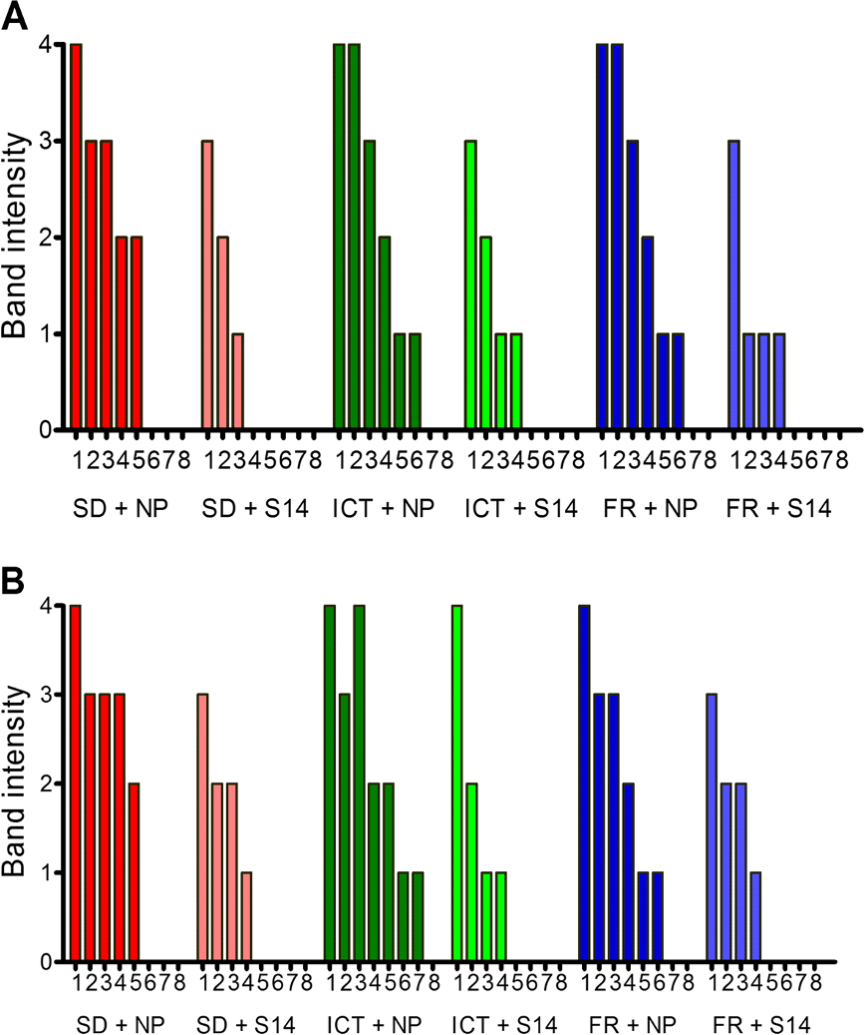
**Anti-PfHRP2 antibody impairs the ability of three PfHRP2-detecting RDTs to detect cultured** ***P. falciparum*** **.** Three brands of RDT (SD, ICT and First Response) were tested with *P. falciparum*-parasitized red cells pre-incubated with normal plasma (NP) or plasma from a subject with high titre anti-PfHRP2 antibody level (S14). Blood was serially diluted using normal human blood maintaining a 50% haematocrit to parasite densities of 100,000, 30,000, 10,000, 3,000, 1,000, 300, 100, and 30 parasites/μL (1–8). The experiment was undertaken before **(A)** and after **(B)** the blood was washed to remove any residual PfHRP2 present in the culture medium.

Suspensions of three separate *P. falciparum* lines were tested at seven different dilutions (100,000 P/μL to 30 P/μL) by incubating each with either a human plasma sample of high anti-PfHRP2 antibody (S14) or normal human plasma for one hour at room temperature, then tested in the antigen ELISA and the three RDTs named above. When the three brands of RDT were tested, a varying level of blocking effect was observed for all three brands tested against the N70 parasite line that originated from the Solomon Islands (Figure 6). The effect was apparent as a reduction of signal intensity from four to three or two at 100,000 P/μl and/or a reduction of lower detection threshold by ten-fold (from 300 to 3,000 P/μl, or from 1,000 to 10,000/μl). A similar blocking effect was alsoobserved when the three RDTs were tested on S55 line (Additional file 3). However, a markedly lesser blocking effect was seen when RDTs were tested on SJ15 line (Additional file 4).

## Discussion

PfHRP2-detecting RDTs are the most important tools for the rapid diagnosis of falciparum malaria and their results form the basis for case management decisions. Factors that may influence the accuracy and sensitivity of PfHRP2-detecting RDTs therefore need to be investigated and their potential impact evaluated. The purpose of this study was to investigate the potential for anti-PfHRP2 antibody accumulated in semi-immune individuals, to influence the performance of RDTs in detecting blood stage *P. falciparum* parasites.

The results of this study indicate that detectable anti-PfHRP2 antibody was present in up to 35% of patients presenting with *P. falciparum* infection in three countries from Africa, Asia and Oceania, with an overall prevalence of 25%. Anti-PfHRP2 antibody was also detected in 10.7% of asymptomatic Solomon Islanders, including individuals without parasitaemia detected by blood smear, suggesting that they carried pre-formed anti-PfHRP2 antibodies. Although antibody levels did not correlate with age or incident parasitaemia among the subjects, the highest antibody levels were observed in a Solomon Islander and Nigerian who lived in countries with areas of high transmission intensity, compared to subjects from the Cambodia and the Philippines where transmission intensity is generally lower. This implies that the antibody levels may relate to transmission intensity, i.e. the rate of exposure to infections. However, the relatively low prevalence of antibodies in people living in high transmission areas of Solomon Islands, the lack of correlation between antibody levels and age or parasitaemia suggest that PfHRP2 is likely not highly immunogenic.

As expected, PfHRP2 antigen was also detected in plasma of most of the patients with acute *P. falciparum* infection from three countries. A subset of these samples (n = 42; 14, 19 and nine from Cambodia, Nigeria and the Philippines, respectively) had previously been tested positive (100%) for PfHRP2 antigen in the whole blood sample using an in-house ELISA. Forty of these 42 plasma samples were also positive for PfHRP2 by ELISA. The negative test results for PfHRP2 antigen in the plasma of the two patients in Nigeria who had moderate levels of parasitaemia (49,855/μl and 6,815/μl) and detectable antigen in whole blood is unexpected and requires further investigation. One possibility is that in early infection the levels of free antigen in plasma are below the level of detection, as it is known that the majority of PfHRP2 is present within infected red cells and released into plasma when infected erythrocytes are destroyed [18]. It is also possible that the PfHRP2 had degraded during storage and transport of the two plasma samples. Antibodies to PfHRP2 were present in the plasma of the subject with a parasitaemia of 49,855 but not in the other. Thus, antibody binding could only explain one case.

Of note, a significant positive correlation between parasite density and plasma PfHRP2 antigen level was detected. The level of PfHRP2 in patients in a high transmission area (Nigeria) was significantly higher than that in patients from a low transmission area (the Philippines). This may be related to differences in the level of acquired immune responses between the subjects of these two countries. People living in areas with low transmission intensity such as the Philippines, are more likely to have less immunity and present for treatment early during an infection. These people would be expected to have lower level of PfHRP2 in their plasma than those from Nigeria. It is also possible that this observation is due to sampling bias as the study was not designed to systematically answer the question of the association between antigenaemia, antibody levels and levels of transmission.

It is of interest that subjects with high plasma antigen levels tended to have low antibody level and *vice versa*, suggesting antibody-antigen complexes may have been formed in patients’ plasma. These immune complexes are expected to not only impact on the prevalence and levels of anti-PfHRP2 in the subjects, but more importantly, to block the binding of free-PfHRP2 by antibodies embedded in malaria RDTs, potentially leading to false negative results. This effect has been previously reported in antigen-capture diagnostic tests, for example with p24 antigen in HIV [30]. In this setting, harsh methods not readily amenable to adaptation to card tests (such as acid [30] or heat [31] dissociation) were required to dissociate these immune complexes. In this study the effect of dissociation of immune complexes as a means of abrogating this effect was not investigated. Likewise, the explanation for one of the tests being less affected was not explored, and remains a priority for future study. It is possible however that the single brand of RDT for which a blocking effect was not detected may have incorporated a step that would achieve this objective. It also remains possible that this blocking effect is caused by a phenomenon other than pre-existing antibodies as the role of such antibodies has not been conclusively established in this study.

In this study the pre-incubation of plasma with high titre antibody (16.7-fold higher than the cut-off value) against PfHRP2 with culture supernatant containing soluble PfHRP2 resulted in a significant reduction in OD values of ELISA and a false negative reading on two of the three RDTs tested. This effect could be titrated out by diluting the plasma 1:5. When the same plasma was incubated with cultures containing intact parasitized erythrocytes, a reduction in band intensity at the highest parasite density was observed for all three brands of RDTs. Importantly, unlike soluble antigens, no false negative RDT results were observed at the highest parasite density. However, the lower detection threshold decreased by ten-fold in all three brands of RDTs tested on two of the three parasite lines. The relatively weaker blocking effect observed in the detection of whole cell compared to soluble antigens is likely due to less free PfHRP2 available to form immune complex at the pre-incubation as most of PfHRP2 is located within the red cells. The majority of intra-erythrocytic PfHRP2 would only be released when blood cells are lysed on RDTs. Even so, a proportion of PfHRP2 was able to form immune complex with antibodies in the plasma immediately upon the lysis of blood on the RDT, and to reduce signal intensity at highest parasite density, and to reduce the lower detection threshold by ten-fold. This is supported by a similar blocking effect observed when parasite cultures were washed with fresh media to remove free PfHRP2.

The findings of this study confirm that people infected with *P. falciparum* parasites develop antibodies against PfHRP2, with some subjects developing very high levels of anti-PfHRP2 antibody. As a consequence, the detection sensitivity of RDTs on blood samples from individuals with such anti-PfHRP2 antibodies can fall by up to ten-fold. This has important implications for malaria diagnosis in areas with high transmission where people are often in semi- to hyper-immune, and have high-titre antibodies to a broad range of parasite antigens. Like the prozone-like effect described elsewhere [12, 13], this would limit the ability to rely on RDT test line intensity (or an electronic reader) to determine parasite load and severity of infection. The antibody-antigen interaction and its potential impact on RDT performance are illustrated in Additional file 5.

The impact of antibody blocking on the detection sensitivity of RDTs will depend on the prevalence and the titre of anti-PfHRP2 antibodies. In the relatively small sample sets from the Solomon Islands (n = 28) and Nigeria (n = 23), one individual in each group had very high antibody titre (>five-fold higher than the cut-off value), and co-incubation of these sera had a significant effect on the detection threshold of RDTs. The effect of antibody blocking may be increased if saliva [32] or urine [33] are tested for soluble PfHRP2, as levels are lower in these biological fluids, and secreted antibody may be present. It is also possible that a similar blocking effect may affect the performance of RDTs targeting parasite biomarkers other than PfHRP2, such as LDH and aldolase. It should be noted that this is a proof of concept study demonstrating that individuals with antibodies against HRP2 can block the RDT detection of parasites, and future studies are required to define the minimum level of antibodies required to cause a significant blocking effect.

## Conclusion

Although further studies are required to determine the sero-epidemiology of anti-PfHRP2 antibodies in areas with different transmission intensities, as well as a more comprehensive investigation of this factor as a contributor to false negative tests, these observations indicate possible reduced diagnostic sensitivity for diagnosis of *P. falciparum* malaria using PfHRP2-detecting RDTs, particularly among people with high levels of specific antibodies and low density infection.

## Electronic supplementary material

Additional file 1: **Parasite densities in subjects with acute** ***Plasmodium falciparum*** **infection.** Country of origin is represented using ISO 3166 country code: Cambodia (KH), Nigeria (NG), the Philippines (PH) and Solomon Islands (SB). Bars represent geometric mean with 95% CI. (PDF 21 KB)

Additional file 2: **Optical Density values of PfHRP2-specific antibody levels in the plasma of subjects measured by ELISA.** Horizontal lines denote the mean OD in each group. Country of origin is represented using ISO 3166 country code: Cambodia (KH), Nigeria (NG), the Philippines (PH), and Solomon Islands (SB). BNE represents Brisbane. Samples from subjects who were microscopy negative for *P. falciparum* are shown as Neg. (PDF 25 KB)

Additional file 3: **Anti-PfHRP2 antibody impairs the ability of three PfHRP2-detecting RDTs to detect cultured** ***P. falciparum*** **S55.** Three brands of RDT (SD, ICT and First Response) were tested with *P. falciparum*-parisitized red cells pre-incubated with normal plasma (NP) or plasma from a subject with high titre anti-PfHRP2 antibody level (S14). Blood was serially diluted using normal human blood maintaining a 50% haematocrit to parasite densities of 100,000, 30,000, 10,000, 3,000, 1,000, 300, 100, and 30 parasites/μL (1-8). The experiment was undertaken before **(A)** and after **(B)** the blood was washed to remove any residual PfHRP2 present in the culture medium. (PDF 149 KB)

Additional file 4: **Anti-PfHRP2 antibody impairs the ability of three PfHRP2-detecting RDTs to detect cultured** ***P. falciparum*** **SJ15.** Three brands of RDT (SD, ICT and First Response) were tested with *P. falciparum*-parisitized red cells pre-incubated with normal plasma (NP) or plasma from a subject with high titre anti-PfHRP2 antibody level (S14). Blood was serially diluted using normal human blood maintaining a 50% haematocrit to parasite densities of 100,000, 30,000, 10,000, 3,000, 1,000, 300, 100, and 30 parasites/μL (1-8). The experiment was undertaken before **(A)** and after **(B)** the blood was washed to remove any residual PfHRP2 present in the culture medium. (PDF 178 KB)

Additional file 5: **Illustration of interactions between host anti-PfHRP2 antibodies and parasite PfHRP2 and their potential impact on RDT performance.** (PDF 112 KB)

## References

[1] WHO (2010). Guidelines for the Treatment of Malaria.

[2] WHO (2013). World Malaria Report 2013.

[3] Stow NW, Torrens JK, Walker J (1999). An assessment of the accuracy of clinical diagnosis, local microscopy and a rapid immunochromatographic card test in comparison with expert microscopy in the diagnosis of malaria in rural Kenya. Trans R Soc Trop Med Hyg.

[4] Wongsrichanalai C (2001). Rapid diagnostic techniques for malaria control. Trends Parasitol.

[5] Endeshaw T, Gebre T, Ngondi J, Graves PM, Shargie EB, Ejigsemahu Y, Ayele B, Yohannes G, Teferi T, Messele A, Zerihun M, Genet A, Mosher AW, Emerson PM, Richards FO (2008). Evaluation of light microscopy and rapid diagnostic test for the detection of malaria under operational field conditions: a household survey in Ethiopia. Malar J.

[6] van den Broek I, Hill O, Gordillo F, Angarita B, Hamade P, Counihan H, Guthmann JP (2006). Evaluation of three rapid tests for diagnosis of P. falciparum and P. vivax malaria in Colombia. Am J Trop Med Hyg.

[7] Rakotonirina H, Barnadas C, Raherijafy R, Andrianantenaina H, Ratsimbasoa A, Randrianasolo L, Jahevitra M, Andriantsoanirina V, Menard D (2008). Accuracy and reliability of malaria diagnostic techniques for guiding febrile outpatient treatment in malaria-endemic countries. Am J Trop Med Hyg.

[8] Wongsrichanalai C, Barcus MJ, Muth S, Sutamihardja A, Wernsdorfer WH (2007). A review of malaria diagnostic tools: microscopy and rapid diagnostic test (RDT). Am J Trop Med Hyg.

[9] Birku Y, Welday D, Ayele D, Shepherd A (1999). Rapid diagnosis of severe malaria based on the detection of Pf-Hrp-2 antigen. Ethiop Med J.

[10] Gamboa D, Ho MF, Bendezu J, Torres K, Chiodini PL, Barnwell JW, Incardona S, Perkins M, Bell D, McCarthy J, Cheng Q (2010). A large proportion of *P. falciparum* isolates in the Amazon region of Peru lack pfhrp2 and pfhrp3: implications for malaria rapid diagnostic tests. PLoS One.

[11] Baker J, Gatton ML, Peters J, Ho M-F, McCarthy JS (2011). Transcription and expression of *Plasmodium falciparum* histidine-rich proteins in different stages and strains: implications for rapid diagnostic tests. PLoS One.

[12] Gillet P, Mori M, Van Esbroeck M, Van den Ende J, Jacobs J (2009). Assessment of the prozone effect in malaria rapid diagnostic tests. Malar J.

[13] Luchavez J, Baker J, Alcantara S, Belizario V, Cheng Q, McCarthy JS, Bell D (2011). Laboratory demonstration of a prozone-like effect in HRP2-detecting malaria rapid diagnostic tests: implications for clinical management. Malar J.

[14] Chiodini PL, Bowers K, Jorgensen P, Barnwell JW, Grady KK, Luchavez J, Moody AH, Cenizal A, Bell D (2007). The heat stability of Plasmodium lactate dehydrogenase-based and histidine-rich protein 2-based malaria rapid diagnostic tests. Trans R Soc Trop Med Hyg.

[15] Lee N, Gatton ML, Pelecanos A, Bubb M, Gonzalez I, Bell D, Cheng Q, McCarthy JS (2012). Identification of optimal epitopes for *Plasmodium falciparum* rapid diagnostic tests that target histidine-rich proteins 2 and 3. J Clin Microbiol.

[16] WHO (2012). Malaria Rapid Diagnostic Test Performance. Results of WHO Product Testing of Malaria RDTs: Round 4.

[17] Howard RJ, Uni S, Aikawa M, Aley SB, Leech JH, Lew AM, Wellems TE, Rener J, Taylor DW (1986). Secretion of a malarial histidine-rich protein (Pf HRP II) from *Plasmodium falciparum*-infected erythrocytes. J Cell Biol.

[18] Desakorn V, Dondorp AM, Silamut K, Pongtavornpinyo W, Sahassananda D, Chotivanich K, Pitisuttithum P, Smithyman AM, Day NP, White NJ (2005). Stage-dependent production and release of histidine-rich protein 2 by *Plasmodium falciparum*. Trans R Soc Trop Med Hyg.

[19] Mayxay M, Pukrittayakamee S, Chotivanich K, Looarecsuwan S, White N (2001). Persistence of *Plasmodium falciparum* HRP-2 in successfully treated acute falciparum malaria. Trans R Soc Trop Med Hyg.

[20] Tjitra E, Suprianto S, Dyer ME, Currie BJ, Anstey NM (2001). Detection of histidine rich protein 2 and panmalarial ICT Malaria Pf/Pv test antigens after chloroquine treatment of uncomplicated falciparum malaria does not reliably predict treatment outcome in eastern Indonesia. Am J Trop Med Hyg.

[21] Noedl H, Wernsdorfer WH, Miller RS, Wongsrichanalai C (2002). Histidine-rich protein II: a novel approach to malaria drug sensitivity testing. Antimicrob Agents Chemother.

[22] Biswas S, Tomar D, Rao DN (2005). Investigation of the kinetics of histidine-rich protein 2 and of the antibody responses to this antigen, in a group of malaria patients from India. Ann Trop Med Parasitol.

[23] Johnson BC, Swanson AM (1952). Milk Serum Proteins. I. A quantitative Biuret test for milk serum proteins. J Dairy Sci.

[24] WHO (2009). Malaria Rapid Diagnostic Tests Performance : Results of WHO Product Testing of Malaria RDTs: Round 1 (2008).

[25] WHO (2010). Malaria Rapid Diagnostic Test Performance Results of WHO Product Testing of Malaria RDTs: Round 2 (2009).

[26] WHO-TDR-FIND (2010). Methods Manual for Laboratory Quality Control Testing of Malaria Rapid Diagnostic Test, Version 6.

[27] Rzepczyk CM, Hale K, Woodroffe N, Bobogare A, Csurhes P, Ishii A, Ferrante A (1997). Humoral immune responses of Solomon Islanders to the merozoite surface antigen 2 of *Plasmodium falciparum* show pronounced skewing towards antibodies of the immunoglobulin G3 subclass. Infect Immun.

[28] Trager W, Jensen JB (1976). Human malaria parasites in continuous culture. Science.

[29] Lambros C, Vanderberg JP (1979). Synchronization of *Plasmodium falciparum* erythrocytic stages in culture. J Parasitol.

[30] Nishanian P, Huskins KR, Stehn S, Detels R, Fahey JL (1990). A simple method for improved assay demonstrates that HIV p24 antigen is present as immune complexes in most sera from HIV-infected individuals. J Infect Dis.

[31] Schupbach J, Flepp M, Pontelli D, Tomasik Z, Luthy R, Boni J (1996). Heat-mediated immune complex dissociation and enzyme-linked immunosorbent assay signal amplification render p24 antigen detection in plasma as sensitive as HIV-1 RNA detection by polymerase chain reaction. AIDS.

[32] Wilson NO, Adjei AA, Anderson W, Baidoo S, Stiles JK (2008). Detection of *Plasmodium falciparum* histidine-rich protein II in saliva of malaria patients. Am J Trop Med Hyg.

[33] Genton B, Paget S, Beck HP, Gibson N, Alpers MP, Hii J (1998). Diagnosis of *Plasmodium falciparum* infection using ParaSight(R)-F test in blood and urine of Papua New Guinean children. Southeast Asian J Trop Med Public Health.

