# Supplementary figures and images for: Circulating antibodies against Plasmodium falciparum histidine-rich proteins 2 interfere with antigen detection by rapid diagnostic tests
Source: Malar J. 2014 Dec 6;13:480. doi: 10.1186/1475-2875-13-480 (PMC4295572; doi:10.1186/1475-2875-13-480)

Parasitemia

1000000  
100000  
10000  
1000  
100  
10

KH (n=15)

NG (n=23)

PH (n=37)

Country of origin

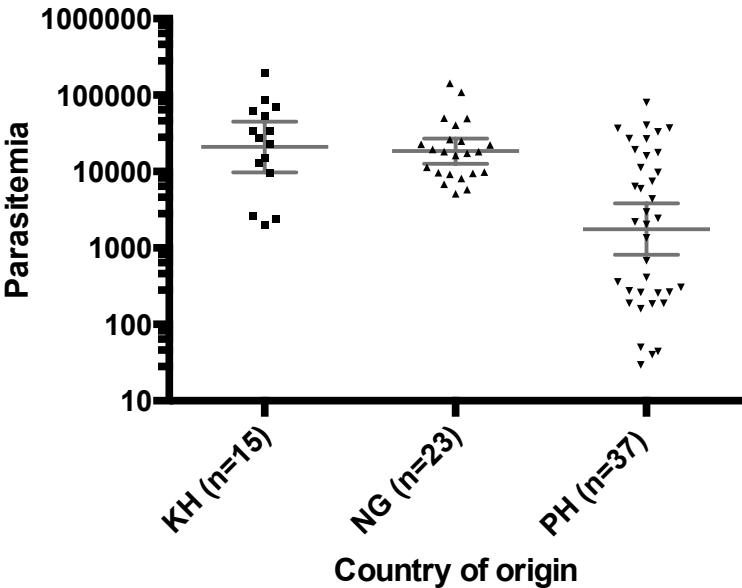

Supplement: Supplementary file 1 — Additional file 1: Parasite densities in subjects with acute Plasmodium falciparum infection. Country of origin is represented using ISO 3166 country code: Cambodia (KH), Nigeria (NG), the Philippines (PH) and Solomon Islands (SB). Bars represent geometric mean with 95% CI. (PDF 21 KB) [file 12936_2014_3633_MOESM1_ESM.pdf]

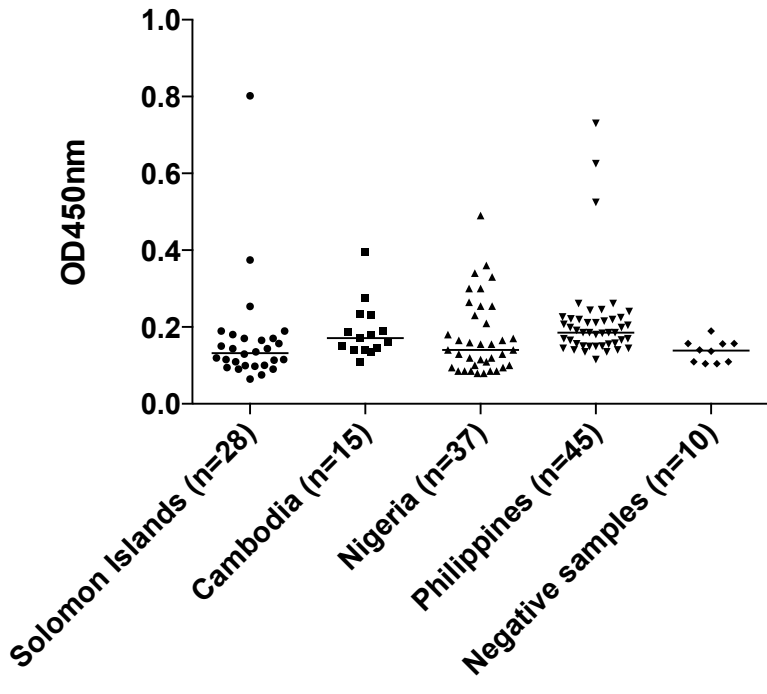

Supplement: Supplementary file 2 — Additional file 2: Optical Density values of PfHRP2-specific antibody levels in the plasma of subjects measured by ELISA. Horizontal lines denote the mean OD in each group. Country of origin is represented using ISO 3166 country code: Cambodia (KH), Nigeria (NG), the Philippines (PH), and Solomon Islands (SB). BNE represents Brisbane. Samples from subjects who were microscopy negative for P. falciparum are shown as Neg. (PDF 25 KB) [file 12936_2014_3633_MOESM2_ESM.pdf]

**A.**

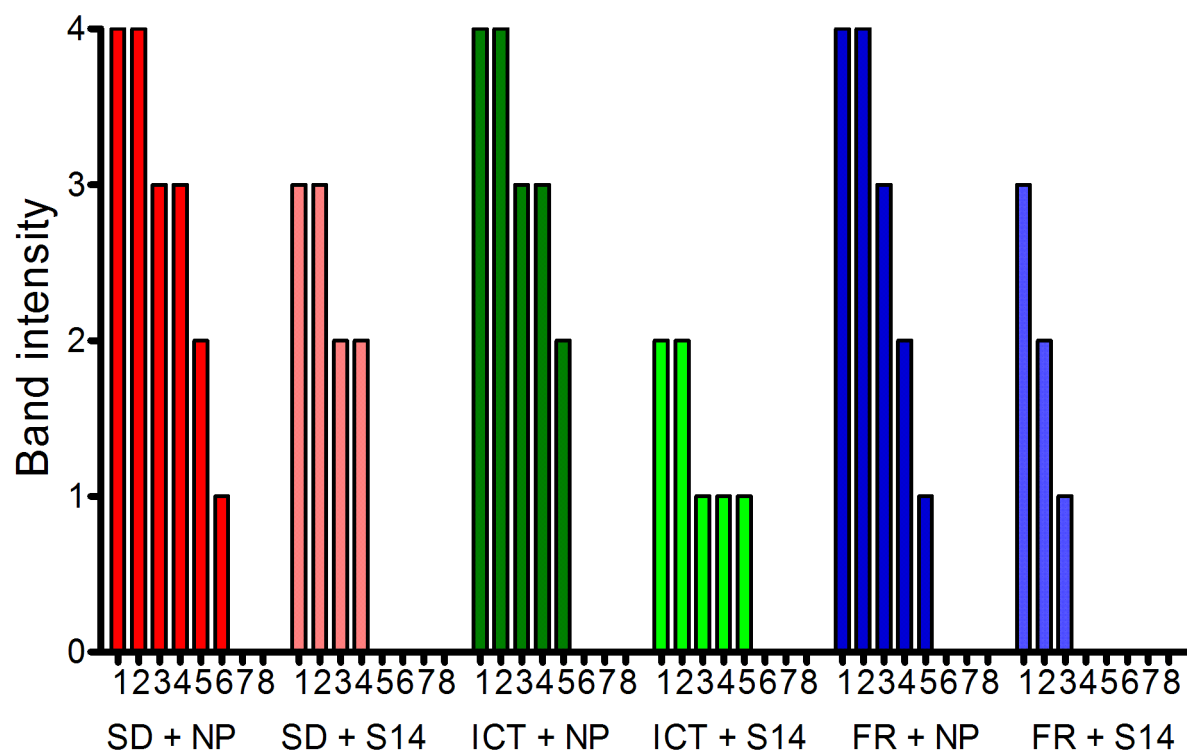

**B.**

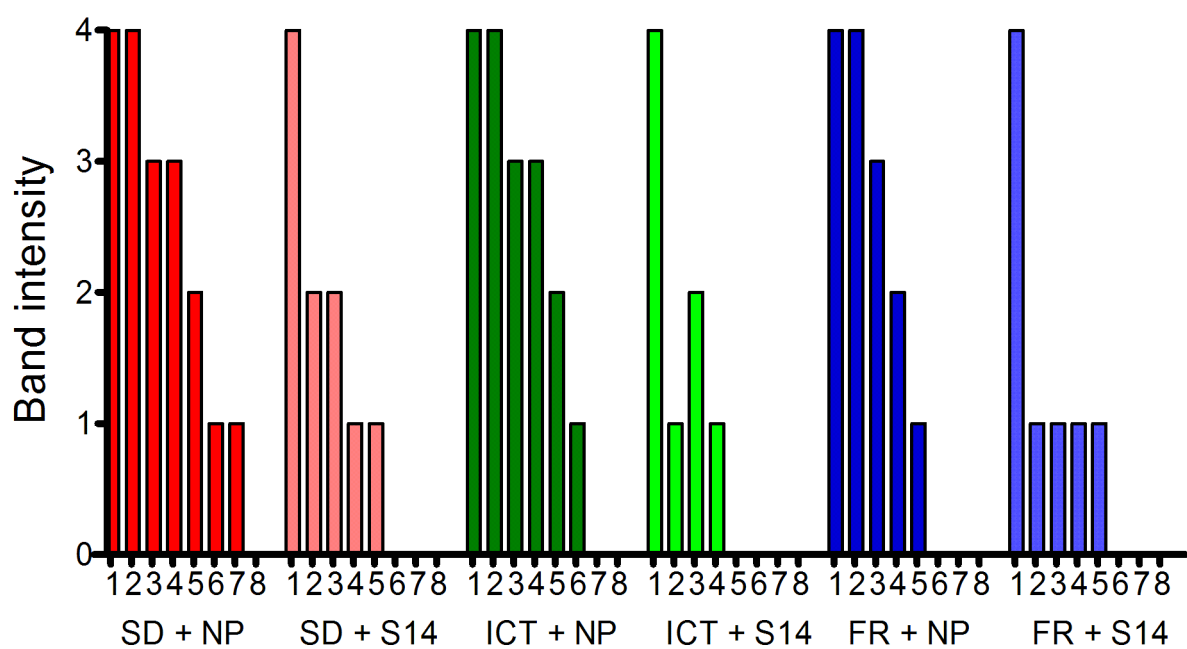

Supplement: Supplementary file 3 — Additional file 3: Anti-PfHRP2 antibody impairs the ability of three PfHRP2-detecting RDTs to detect cultured P. falciparum S55. Three brands of RDT (SD, ICT and First Response) were tested with P. falciparum-parisitized red cells pre-incubated with normal plasma (NP) or plasma from a subject with high titre anti-PfHRP2 antibody level (S14). Blood was serially diluted using normal human blood maintaining a 50% haematocrit to parasite densities of 100,000, 30,000, 10,000, 3,000, 1,000, 300, 100, and 30 parasites/μL (1-8). The experiment was undertaken before (A) and after (B) the blood was washed to remove any residual PfHRP2 present in the culture medium. (PDF 149 KB) [file 12936_2014_3633_MOESM3_ESM.pdf]

**A.**

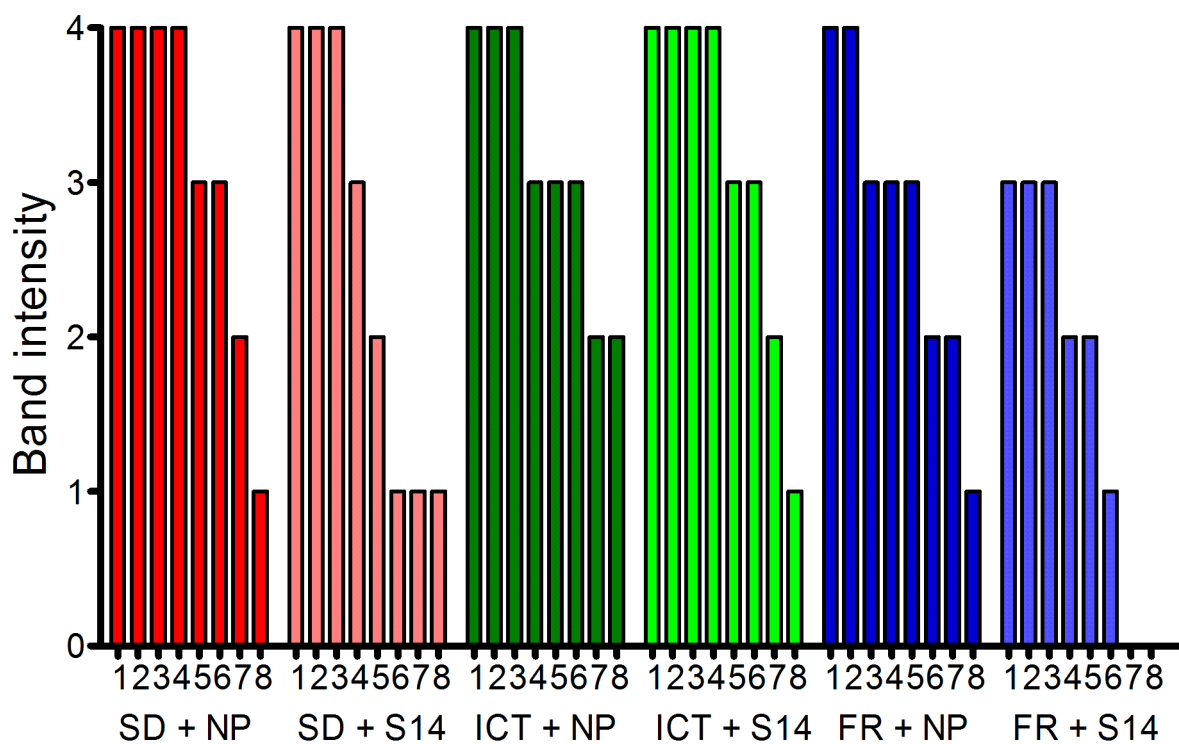

**B.**

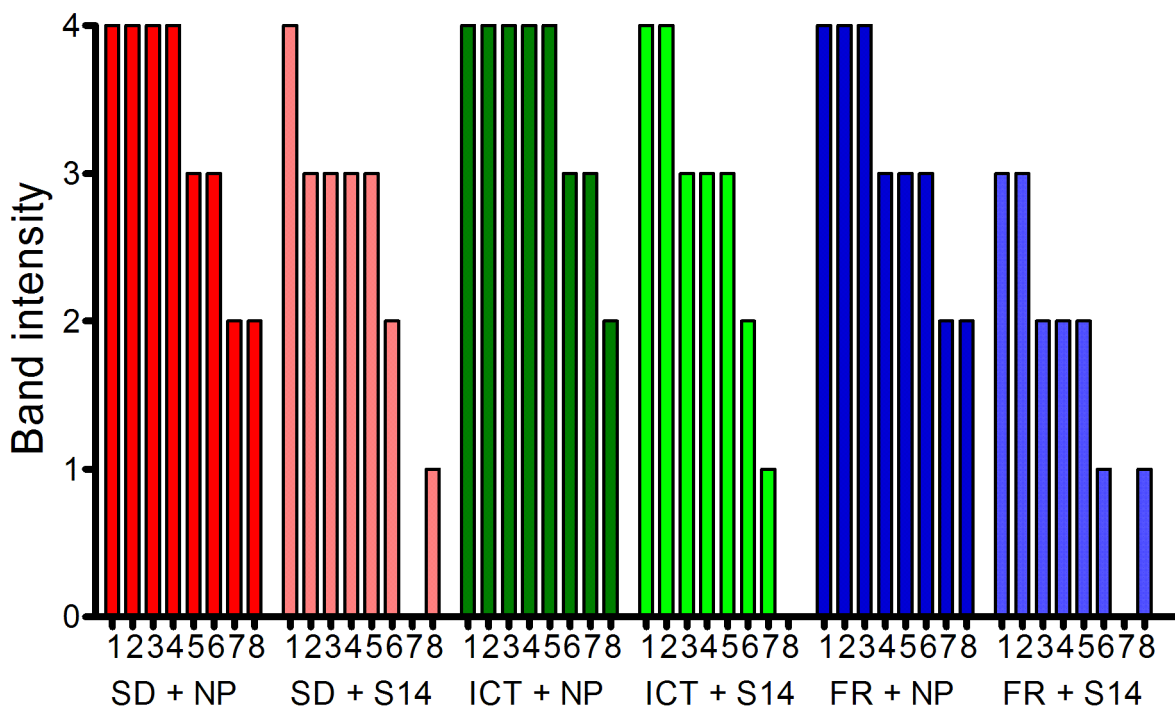

Supplement: Supplementary file 4 — Additional file 4: Anti-PfHRP2 antibody impairs the ability of three PfHRP2-detecting RDTs to detect cultured P. falciparum SJ15. Three brands of RDT (SD, ICT and First Response) were tested with P. falciparum-parisitized red cells pre-incubated with normal plasma (NP) or plasma from a subject with high titre anti-PfHRP2 antibody level (S14). Blood was serially diluted using normal human blood maintaining a 50% haematocrit to parasite densities of 100,000, 30,000, 10,000, 3,000, 1,000, 300, 100, and 30 parasites/μL (1-8). The experiment was undertaken before (A) and after (B) the blood was washed to remove any residual PfHRP2 present in the culture medium. (PDF 178 KB) [file 12936_2014_3633_MOESM4_ESM.pdf]

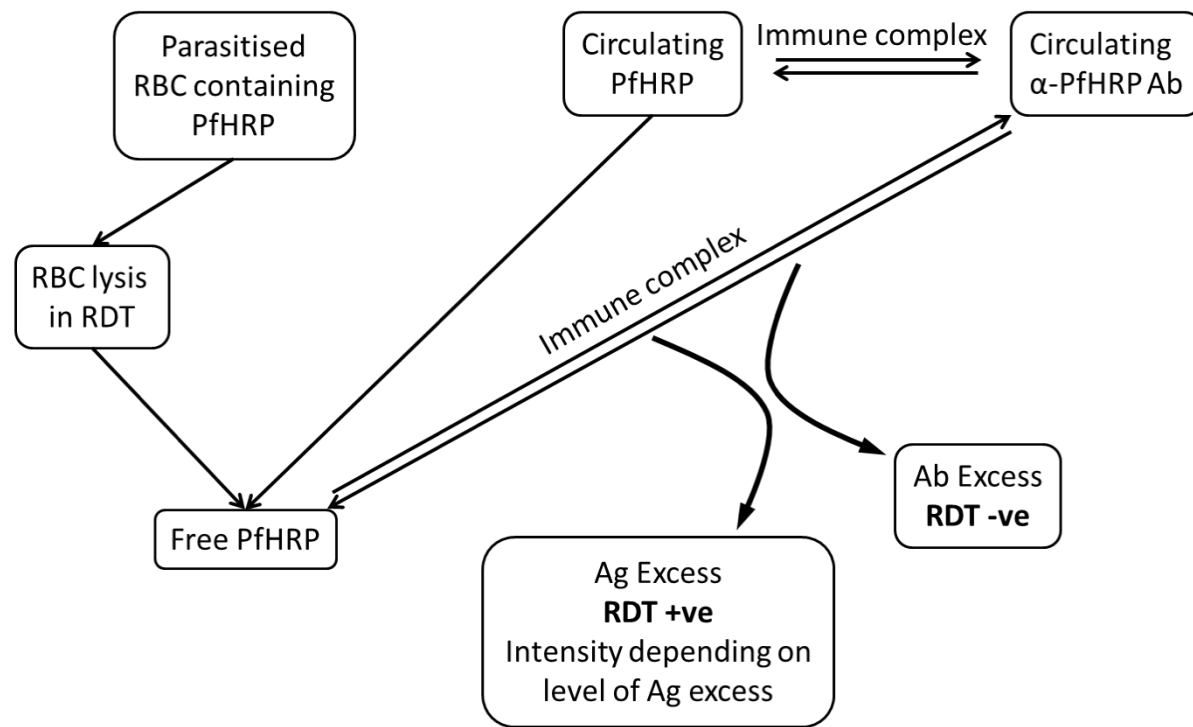

Supplement: Supplementary file 5 — Additional file 5: Illustration of interactions between host anti-PfHRP2 antibodies and parasite PfHRP2 and their potential impact on RDT performance. (PDF 112 KB) [file 12936_2014_3633_MOESM5_ESM.pdf]
